# Self-Certified Sickness Absence among Young Municipal Employees—Changes from 2002 to 2016 and Occupational Class Differences

**DOI:** 10.3390/ijerph14101131

**Published:** 2017-09-26

**Authors:** Hilla Sumanen, Olli Pietiläinen, Minna Mänty

**Affiliations:** 1 Department of Public Health, University of Helsinki, P.O. Box 20 (Tukholmankatu 8B), FIN-00014 Helsinki, Finland; olli.k.pietilainen@helsinki.fi (O.P.); minna.manty@helsinki.fi (M.M.); 2 Department of Health Care and Emergency Care, South-Eastern Finland University of Applied Sciences, FIN-48220 Kotka, Finland; 3 Department of Research, Development and Innovation, Laurea University of Applied Sciences, FIN-01300 Vantaa, Finland

**Keywords:** short-term sick-leave, young adults, socioeconomic differences, gender, municipal employees

## Abstract

We examined changes in self-certified, one-to-three day sickness absence (SA) among young employees from 2002 to 2016 and the magnitude of occupational class differences during that period. All 18–34-year-old employees of the City of Helsinki, Finland were included (2002–2016, *n* = ~11,725 per year). Employer’s personnel and SA registers were used. Occupational class was categorized to four groups. Changes in self-certified SA from 2002 to 2016 were analyzed with Joinpoint regression and the magnitudes of occupational class differences were estimated with the relative index of inequality (RII). Most of the trends first increased and turned to decrease in 2007/2010. Managers and professionals had the least amount of SA, but steadily increasing trends were observed among men. Self-certified SA followed only partially the typical socioeconomic gradient, as routine non-manuals had the highest levels of SA. The magnitude of occupational class differences in self-certified SA was stable during the study period only among women. Self-certified SA and occupational class differences have increased in recent years among men in the lower occupational classes. Socioeconomic differences exist in self-certified SA among young employees, but gradient is only partial. Overall, high amounts of self-certified SA especially in the lower occupational classes require further studies and preventive measures.

## 1. Introduction

Self-certified sickness absence means health-related short-lasting absence from work with an employee’s own notice. In Finland, employees in the municipal sector usually can take up to three self-certified sickness absence days from work. Self-certified sickness absence may represent different causes of absence than longer sickness absence, which requires medical certification. Previous evidence suggests that self-certified sickness absence may reflect employees’ perceptions of their health rather than actual disease, and taking short time off work could be considered a type of coping behavior [1,2]. There is evidence that self-certified sickness absence may be related to motivational issues [3,4] and are potentially used as a self-control over working times [5]. However, from the employer’s viewpoint self-certified sickness absence may cause problems with lost working hours and difficulties in finding replacements at short notice, especially if the sickness absence spells are frequent [6]. Also, previous studies show that frequent self-certified sickness absence predicts longer sickness absence spells, thus they are not trivial for health and work ability [7,8].

Our previous study examined age-differences in sickness absence trends, and showed that sickness absence spells lasting one-to-three days are more common among younger, <35-year-old employees [9]. Several other studies have reported similar findings on negative association between shorter sickness absence and age [10,11,12]. It has been suggested that younger employees might take self-certified sickness absence from work more easily than older employees due to minor health-related complaints, such as headache, fatigue, and nausea, or they just suffer from them more often [13,14,15]. In addition, their recovery may be faster, for example in case of flu, and therefore they do not need to extend their absence with medical certification. However, younger employees might also have negative expectations, attitudes, and values towards work, which may cause them to stay home occasionally [14,15,16,17,18].

Occupational class differences in sickness absence are well studied, and for example, studies from Britain, Japan, Denmark, Sweden, Norway, and Finland have shown a strong hierarchy where the amounts of sickness absences increase with decreasing occupational class [19,20,21,22,23,24,25,26,27]. However, the previous studies have mainly concentrated on older employees and longer sickness absence. There is some evidence that the occupational class gradient might be less clear in shorter than in longer sickness absence [28,29]. Furthermore, little is known about the magnitudes and changes in the occupational class differences over longer periods. The knowledge about younger employees’ sickness absence is generally very limited, but highly needed when designing and implementing preventive interventions. Early prevention is topical, as young employees should be able to continue working for many more decades.

Our previous study showed that self-certified sickness absence trends first increased from 2002 to 2007 among 18–24-year-old women, to 2008 among 25–29 year-old women and to 2010 among 30–34-years-old women. Then the trends decreased towards the end of the study period (2013) [9]. The aim of this study was to examine self-certified sickness absence among the young (18–34-year-old) employees in more detail by focusing on the changes and magnitudes of occupational class differences among both genders during the study period of 2002–2016, and by using two different measures of self-certified sickness absence, that is, spells and days.

## 2. Materials and Methods

### 2.1. Participants

The participants in this study are employees of the City of Helsinki, Finland. Helsinki is the capital of Finland and largest municipal employer with approximately 40,000 employees (~73% women). City of Helsinki’s personnel register was used to obtain individual-level information on the employees’ socio-demographic factors. In this study, all permanently and temporarily employed young, 18–34-year-old female and male employees of the City of Helsinki from the years 2002–2016 were included (Table 1). Those employees with no information on occupational class were excluded (1.7–3.7% per year). The age-group was chosen based on previous knowledge on self-certified sickness absence being particularly common among <35-year-olds [9]. Secondary data retrieved from registers are used in this study. Conventions of good scientific practice, data protection, and information security have been applied. The study was based on registries and thus ethics approval was not required according to Finnish law [30].

### 2.2. Sickness Absence

Employees of the City of Helsinki can take one-to-three days of sickness absence with their own notice and permission from their supervisor. Data on self-certified sickness absence was collected from the employers sickness absence registers. The policies are the same for each employee and did not change during the study period. The registers cover all employees, their work contracts, and sickness absence spells to an accuracy level of one day [31]. Absences considering other than employee’s own health, for example caring for a sick child, were excluded. Consecutive and overlapping sickness absence spells were combined and >3 days of sickness absence were excluded to ensure only short, self-certified spells were chosen to this study. Self-certified spells mean times that employee has taken <3 days sickness absence from work in a year. Self-certified days mean summed-up sickness absence days from those self-certified short absences from work within a year. According to City of Helsinki policies, there are no limits for self-certified sickness absence spells or days per year, but the supervisor should discuss with the employee about her/his work ability after five spells.

### 2.3. Occupational Class

Occupational class was classified to four hierarchical categories based on job titles in the employers personnel register: managers and professionals (such as teachers, physicians), semi-professionals (such as registered nurses, foremen), routine non-manuals (such as practical nurses, child minders, and clerical employees), and manual workers (such as construction workers and cleaners).

### 2.4. Statistical Methods

**Figure 1:**
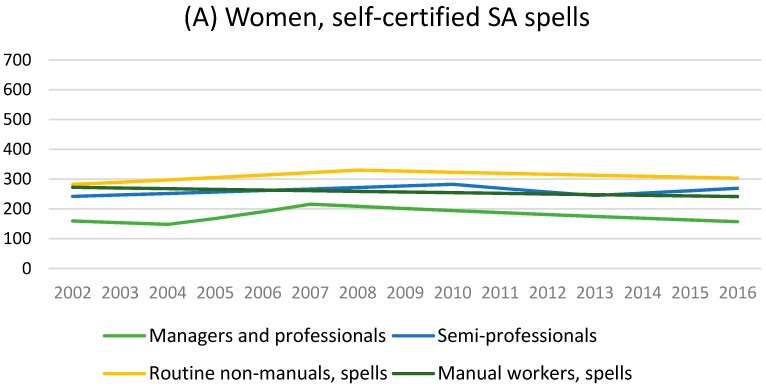

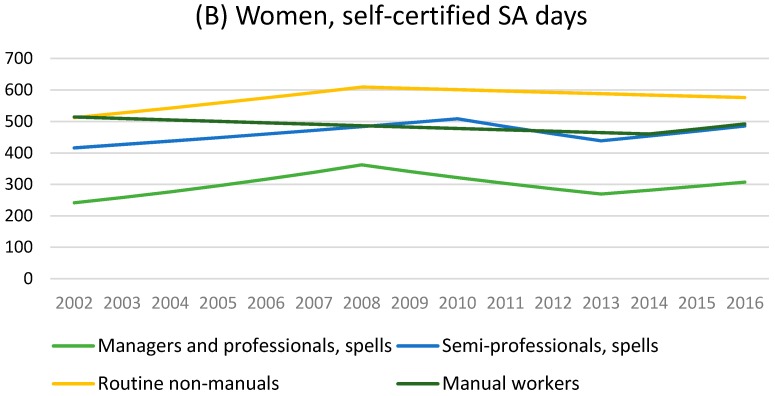
Age-adjusted self-certified sickness absence (SA) spells and days/100 person-years among young women, modelled with Joinpoint regression.

**Figure 2:**
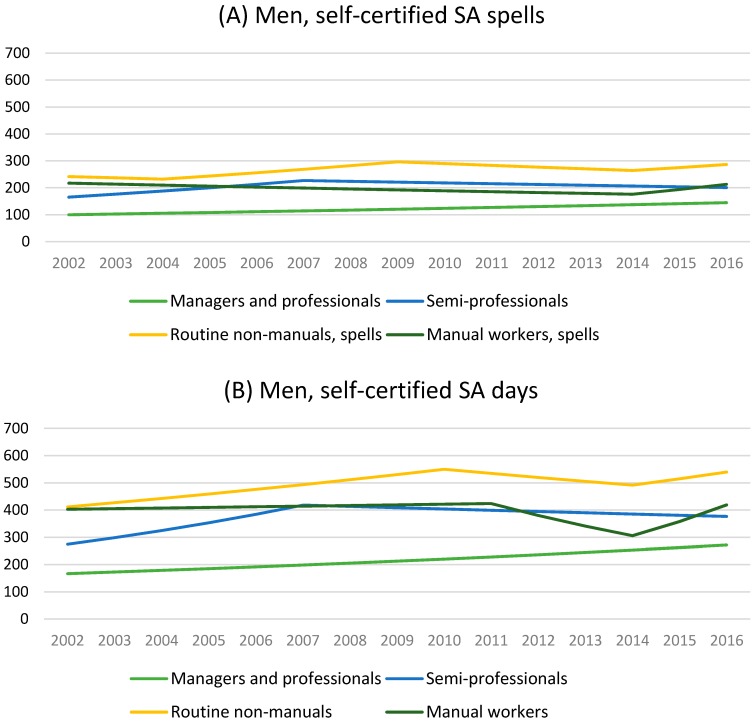
Age-adjusted self-certified sickness absence (SA) spells and days/100 person-years among young men, modelled with Joinpoint regression.

Sickness absences per 100 person-years for self-certified spells and days were calculated annually, i.e., each year is a cross-section for both genders and all occupational classes (Table 1). Women and men were analyzed separately due to differences in sickness absence levels. Age-adjusted Joinpoint regression modelling [32] was used to identify major turning points in sickness absence trends (Table 2, Figure 1 and Figure 2). The Joinpoint modelling starts with 0 joinpoints (linear line) and then tests whether there are turning points which should be added to the model to better represent the actual sickness absence trends. Annual percent changes along with 95% confidence intervals (CI) are presented for each identified period in the sickness absence trends (Table 2). Joinpoint Regression Program version 4.1.1 were used to conduct the analyses [33].

The age-adjusted relative index of inequality (RII) values and their 95% confidence intervals (CI) were calculated annually to determine the magnitude of the relative occupational class differences in self-certified sickness absence spells and days [34]. When calculating RII, first the values of each occupational class group were converted into a relative rank indicator by calculating the midpoint of the cumulative proportion for each occupational class. The rank indicator value 0 represents the theoretical top of the occupational class hierarchy and 1 the theoretical bottom of the occupational class hierarchy. Then the rank indicator was used as a continuous variable in the negative binomial regression models, and the logarithm of the time of employment was used as the offset so that the different lengths of work contracts were taken into account. The resulting RII values can be interpreted as the rate ratio of having self-certified sickness absence at the bottom compared to the risk at the top of the occupational class hierarchy. IBM SPSS version 22 (IBM Corp., Armonk, NY, USA) was used to calculate RII values.

## 3. Results

Among women, around half were routine non-manual employees, and about one-quarter were semi-professionals (Table 1). Among men, manual workers and routine non-manual employees were the largest groups.

### 3.1. Changes in the Sickness Absence Trends

Among women, managers and professionals had the least amount and routine non-manual employees the largest amount of self-certified sickness absence spells and days during the study period of 2002–2016 (Table 1, Figure 1). With regard to change in self-certified spells, manual workers had slightly decreasing trend during the whole study period (−0.9%, 95% CI −1.7, 0.0 annually), while other groups had first increase and then decrease in their sickness absence trends (Table 2). Semi-professionals and routine non-manual employees had statistically significant increase in sickness absence spells until 2010 (2.0%, 95% CI 0.4, 3.5 annually) and 2008 (2.7%, 95% CI 0.9, 4.5 annually), respectively. Strongest decrease from 2007 to 2016 was among managers and professionals (−3.5%, 95% CI −5.7, −1.2). In case of change in self-certified days, the statistically significant turning points were located between 2008–2010, and the strongest increase from 2002 to 2008 was among managers and professionals (7.0%, 95% CI 0.3, 14.2 annually).

Among men, managers and professionals had the least amount and routine non-manuals the highest amount of self-certified sickness absence spells and days (Table 1, Figure 2). Managers and professionals had steadily increasing trends (2.7%, 95% CI 0.0, 5.4 annually in case spells and 3.5%, 95% CI 1.0, 6.2 annually in throughout the study period (Table 2). The trends among semi-professionals were not statistically significant. Among routine non-manuals, the trends increased until 2009/2010 (from 2004 to 2009 5.0%, 95% CI 3.6, 6.4 annually in spells and 3.7%, 95% CI 2.0, 5.4 in days). There was some tendency shown that the trends have turned to increase in the recent years among routine non-manuals (from 2014 to 2016 4.1%, CI 0.5, 7.9 annually) and manual workers (from 2014 to 2016 17.1%, CI 0.6, 36.3 annually).

### 3.2. The Magnitude of Socioeconomic Differences

**Figure 3:**
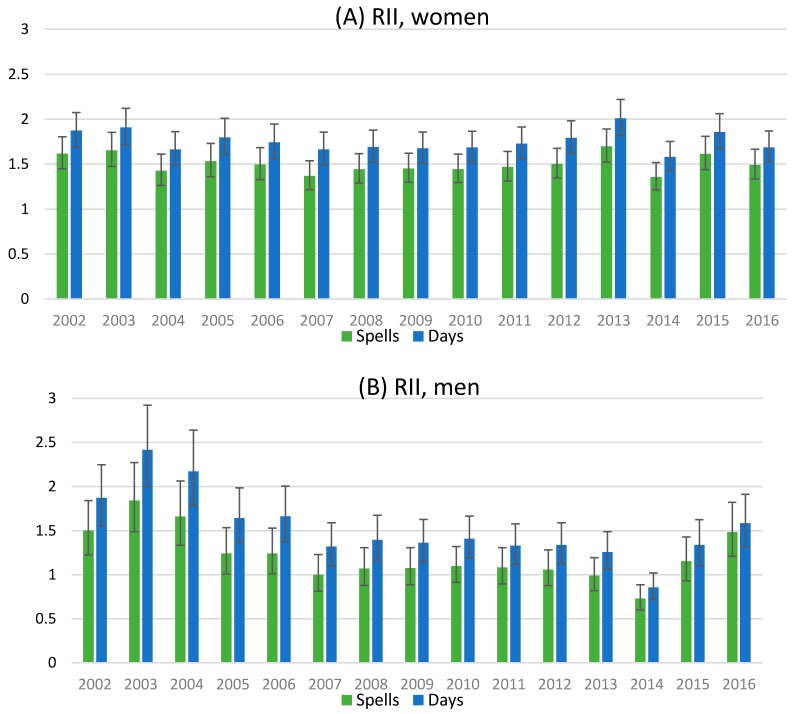
The relative index of inequality (RII) for self-certified sickness absence spells and days according to occupational class, adjusted for age.

Among women, the relative occupational class inequalities as measured by Relative Index of Inequality (RII) were similar in spells and days (Figure 3). The RII values show that those in the hypothetical bottom have broadly 1.5 times more spells and days compared to those in the hypothetical top. Among men there was more annual variation. At the beginning of the study period the RII values were approximately 1.5–2, and then from 2007 to 2013 close to 1, which implies no difference. In 2014 those in the bottom had less self-certified sickness absence than those in the top according to RII values. Since 2015, the relative differences have increased among men.

## 4. Discussion

We examined how self-certified, one-to-three day sickness absences has changed from 2002 to 2016 among young, 18–34-year-old female and male employees in four occupational classes. We also examined the magnitude of occupational class differences. Our main results were: (1) Self-certified sickness absence trends varied during the study period, most of the trends had a turning point to decrease in 2007/2010; (2) Managers and professionals had the least amount of self-certified sickness absence, but there was a steadily increasing trend among men; (3) Self-certified sickness absence does not fully follow the typical socioeconomic gradient; (4) The magnitude of occupational class differences in self-certified sickness absence was stable among women, but changed during the study period among men; (5) There were some indications that occupational class differences have increased in recent years among men in the lower occupational classes.

Changes in the trends were broadly similar in terms of spells and days. This implies that the self-certified spells have been broadly same length from year to year. In our previous study among young women, the turning points in the sickness absence spell trends were placed in the year 2008 [28] for each occupational class. In this study with a longer study period such turning point was found only among routine non-manuals. Since 2008, there has been yearly variation in the sickness absence levels, and thus the turning points were less clear and varied more by occupational class group in this study. Male managers and professionals had increasing trends throughout the study period, but still least amount of sickness absence from all studied groups. Changes in the economic cycles are likely to explain part of the variation in the sickness absence trends. The economic downturn started around 2008 in Finland. Previous studies have shown that sickness absence is procyclical and sickness absence trends increase in the periods of economic boom, when jobs are secured, and vice versa [35]. The turning points found in this study support these previous findings. In addition, we found some indications of increasing amounts of self-certified sickness absence during recent years. These are also in line with the first marks of recovering economy in Finland [36].

Socio-economic differences in sickness absence are well established and previous studies show that those in the lower socioeconomic positions have a higher risk for sickness absence than those in the higher positions [37,38,39,40]. Our results show that the socioeconomic gradient is not fully clear in self-certified sickness absence among young female and male employees. The routine non-manuals had more sickness absence than the manual workers and in some years more than semi-professional employees among both genders. Unusual socioeconomic pattering in self-certified sickness absence has been reported also in a Danish study with hospital staff [29]. In that study, cleaners and porters were the lowest socioeconomic group and had a lower risk of one-to-three day sickness absence spells than the highest socioeconomic group of doctors. This type of socioeconomic structure in shorter absences might be related to lowest occupational classes exceeding other classes in case of longer absences (total sickness absence days) [29,40] and all-length spells [41]. This would mean that the diseases responsive of the absence would be more severe in the lowest class.

The magnitude of occupational class difference in self-certified sickness absence was quite steady during the study period among women, but had ups and downs among the men. The RII values take into account the relative sizes of the groups, and show the relative difference compared to those in the hypothetical bottom to those in the top [34]. The magnitudes can be expected to be larger if manual workers had actually more sickness absence than the other, higher occupational classes. Among women the low levels of sickness absence among managers and professionals compared to others is probably the reason for the RII values, indicating 1.5 times more sickness absence to those in the bottom compared to top. Among men, the occupational class differences were steeper in the beginning and almost non-existing at the middle of the study period, and growing again during recent years. However, among young male employees the higher occupational classes are less represented, and thus, the individual characteristics and sickness absence behavior might be more easily reflected as changing magnitude of occupational class differences. In previous studies, socioeconomic differences in sickness absence have been more pronounced among men than women [21,26,27,37,40], but in this study this cannot be concluded.

A Norwegian study [42] with data from four Nordic countries showed strong evidence that health inequalities between socioeconomic groups are already visible among young adults, and health interventions are important particularly in early adulthood. Also, previous results imply that the socioeconomic gradient in health will not be strongly reduced in the near future [42]. In our study, especially among men in the lower occupational classes, there was evidence of a recent increase in self-certified sickness absence, and also increasing socioeconomic differences when comparing lower classes to higher ones. These classes represent roughly 70% of the young men working for City of Helsinki, thus such increase in absence is likely to affect large amount of employees and workplaces. However, female employees in lower classes have higher amounts of self-certified spells and days, suggesting that preventive measures should be targeted equally to these groups.

It is important to notice that self-certified sickness absence is quite common among young employees. On average, one female employee has two-to-three self-certified sickness absence spells and three-to-six self-certified days per year, men slightly less. The employees receive full salary during one-to-three day sickness absences in Finland. A recent study [43] based on middle-aged employees of the City of Helsinki found out that each day of absence causes approximately 130 euros of salary loss to the employer, not including employer’s social security costs or other direct and indirect costs. The salary costs are probably less in case of younger employees without experience bonuses, but still the amount is considerable. Still, self-certified sickness absence may contribute to lower medical costs, as the employee does not need to seek the GP for a sickness absence certificate. However, many young employees work in the field of health care and social services, such as children’s day-care, where it is necessarily to use replacements, thus adding costs and supervisor’s efforts [6]. It should be also noted that self-certified sickness absence may be used for infection prevention and control especially in the health care occupations, where infecting others must be avoided. Previous evidence suggests that self-certified sickness absence among young employees may be related to other possible things beside health [3,4,14,15,44,45]. Thus, the reasons behind self-certified sickness absence should be more closely monitored. Preventive measures should be started at an early age to avoid accumulation of health and other problems later in life, as age- and exposure-related disabilities and illnesses may occur over time.

### Methodological Considerations

The registers used in this study are reliable and complete, as they are kept by the employer and are used as a base of salary payments. Thus there were no missing data. The sickness absence policies are same to all of the employees of the City of Helsinki, and register-marks are made consistently. Employees can be absent due to health causes with their own notice, but some of them still get medical certification, however this is irrelevant in terms of our aims.

The number of employees in our study did not allow studying young employees in smaller age-groups, as the amounts of employees in the higher occupational classes are small among the youngest persons. Also, a small amount of semi-professionals among men probably affected the statistical power.

Using occupational class as the socioeconomic indicator among young employees might be better choice than for example, education. Even 18-year-olds have occupational class (might not be permanent), but perhaps not yet finished education. The final occupational class might be achieved before formal education, as it is possible to work for example as a fixed-term employee before educational qualification.

However, as the sickness absence policies differ between countries and sometimes even across employment sectors (public/private), our results can be generalized with caution only to the Finnish municipal sector.

## 5. Conclusions

Most of the self-certified sickness absence trends first increased and then decreased since 2007/2010. Among men in lower occupational classes, there were indications that the amount of self-certified sickness absences has increased since 2014, which should be noticed in terms of preventive measures. The occupational class differences followed only partially the typical socioeconomic gradient, and future studies should confirm if this is typical among young municipal employees and what are the reasons behind this sickness absence structure. Among women, the magnitude of occupational class differences remained quite stable during the 15-year period, but among men there were changes that should be more closely monitored. Clear occupational class differences can be seen already among young employees, thus implementing measures to narrow down these differences should be started at young age. Overall, young employees have quite a lot of self-certified sickness absence spells and days, and reasons for this should be examined in order to find the right preventive measures to implement. Young employees in the lower occupational classes are in need of extra attention in terms of maintaining work ability.

## Figures and Tables

**Table 1:** Descriptive statistics of the study population by occupational class among 18–34-year-old employees in 2002, 2009, and 2016.

		Year		
		2002	2009	2016
Women, *n*	All	8789	9225	8926
	Managers and professionals, %	12.1	12.7	11.5
	Semi-professionals, %	23.7	23.0	30.2
	Routine non-manuals, %	51.0	52.3	48.9
	Manual workers, %	13.1	12.1	9.4
Women, SA * spells	Managers and professionals	146	187	175
(/100 person-years)	Semi-professionals	230	249	253
	Routine non-manuals	278	319	307
	Manual workers	275	270	258
Women, SA * days	Managers and professionals	237	313	295
(/100 person-years)	Semi-professionals	401	450	455
	Routine non-manuals	507	591	578
	Manual workers	518	523	490
Men, *n*	All	2742	3065	2743
	Managers and professionals, %	13.9	13.6	14.9
	Semi-professionals, %	13.5	12.7	17.8
	Routine non-manuals, %	31.4	32.0	36.2
	Manual workers, %	41.3	41.7	31.2
Men, SA * spells	Managers and professionals	97	120	124
(/100 person-years)	Semi-professionals	176	191	203
	Routine non-manuals	236	283	281
	Manual workers	198	191	218
Men, SA * days	Managers and professionals	160	209	223
(/100 person-years)	Semi-professionals	304	355	369
	Routine non-manuals	427	526	531
	Manual workers	389	405	419

* SA = sickness absence.

**Table 2:** Identified periods (turning points) and annual change (%) in age-adjusted self-certified sickness absence spells and days/100 person-years by occupational class and gender.

	Women		Men	
	Identified Periods (Turning Points)	Annual % Change (95% CI)	Identified Periods (Turning Points)	Annual % Change (95% CI)
**Self-certified sickness absence spells**				
Managers and professionals	2002–2004 2004–2007 2007–2016	−3.7 (−26.3, 25.9) 13.4 (−11.0, 44.6) −3.5 (−5.7, −1.2)	2002–2016	2.7 (0.0, 5.4)
Semi-professionals	2002–2010 2010–2013 2013–2016	2.0 (0.4, 3.5) −4.6 (−15.4, 7.5) 3.2 (−2.9, 9.6)	2002–2007 2007–2016	6.5 (−4.2, 18.5) −1.4 (−5.1, 2.6)
Routine non-manuals	2002–2008 2008–2016	2.7 (0.9, 4.5) −1.1 (−2.1, 0.0)	2002–2004 2004–2009 2009–2014 2014–2016	−2.0 (−6.2, 2.4) 5.0 (3.6, 6.4) −2.3 (−3.4, −1.2) 4.1 (0.5, 7.9)
Manual workers	2002–2016	−0.9 (−1.7, 0.0)	2002–2014 2014–2016	−1.7 (−2.7, −0.8) 10.0 (−10.0, 34.5)
**Self-certified sickness absence days**				
Managers and professionals	2002–2008 2008–2013 2013–2016	7.0 (0.3, 14.2) −5.7 (−15.0, 4.6) 4.5 (−14.7, 28.0)	2002–2016	3.5 (1.0, 6.2)
Semi-professionals	2002–2010 2010–2013 2013–2016	2.5 (1.2, 3.9) −4.8 (−13.9, 5.2) 3.5 (−1.7, 8.8)	2002–2007 2007–2016	8.8 (−2.5, 21.3) −1.2 (−4.9, 2.8)
Routine non-manuals	2002–2008 2008–2016	2.9 (1,2, 4,7) −0.7 (−1,7, 0,3)	2002–2010 2010–2014 2014–2016	3.7 (2.0, 5.4) −2.8 (−8.8, 3.6) 4.8 (−7.6, 18.9)
Manual workers	2002–2014 2014–2016	−0.9 (−2.0, 0.1) 3.4 (−16.4, 28.0)	2002–2011 2011–2014 2014–2016	0.6 (−0.5, 1.7) −10.3 (−20.1, 0.7) 17.1 (0.6, 36.3)
